# Do work ability and life satisfaction matter for return to work? Predictive ability of the work ability index and life satisfaction questionnaire among women with long-term musculoskeletal pain

**DOI:** 10.1186/s12889-021-10510-8

**Published:** 2021-03-24

**Authors:** Mamunur Rashid, Marina Heiden, Annika Nilsson, Marja-Leena Kristofferzon

**Affiliations:** 1 Centre for Musculoskeletal Research, Department of Occupational Health Sciences and Psychology, Faculty of Health and Occupational Studies, University of Gävle, SE-80176 Gävle, Sweden; 2 Department of Caring Sciences, Faculty of Health and Occupational Studies, University of Gävle, Gävle, Sweden

**Keywords:** Musculoskeletal disorders, Quality of life, Sickness absence, Work capacity, Work status

## Abstract

**Background:**

Impaired work ability and reduced life satisfaction due to long-term musculoskeletal pain, particularly in neck, shoulders and back, are considered occupational health problems that can result in workers taking sick leave. The aim of the study was to determine whether work ability and life satisfaction predict return to work (RTW) among women with long-term neck/shoulder and/or back pain, and to assess the ability of the Work Ability Index (WAI) and the Life Satisfaction Questionnaire (LiSat-11) to discriminate between those who did RTW and those who did not RTW (NRTW).

**Methods:**

This is a cohort study with 1-year follow-up. A survey was sent to 600 women receiving sick leave benefits from the Swedish Social Insurance Agency. In total, 208 women responded at baseline, and 141 at a 1-year follow-up. To identify whether work ability and life satisfaction predicted RTW, multiple logistic regression analyses were performed with and without adjustment for type of work and pain intensity. To assess the discriminative ability of the WAI and the LiSat-11 for women who did RTW and those who did NRTW, receiver operating characteristic curves were fitted.

**Results:**

Work ability predicted RTW, and the results remained significant after adjusting for type of work and pain intensity (OR 1.12, 95% CI: 1.04–1.22). Life satisfaction was not significant. The WAI at baseline adequately discriminated between RTW and NRTW after 1 year (Area under curve 0.78, 95% CI: 0.70–0.86), but the LiSat-11 did not.

**Conclusions:**

This study supports a relationship between work ability and RTW among women on sick leave for long-term neck/shoulder and/or back pain. The results indicate that the WAI, but not the LiSat-11, can discriminate between RTW and NRTW in the population under study. Although the discriminative ability of the WAI needs to be verified in new samples before it can be recommended for use in rehabilitation settings, we suggest that healthcare professionals consider how women perceive their work ability in order to better support them in their RTW.

## Background

Impaired work ability and reduced life satisfaction due to long-term musculoskeletal pain (MSP) in the neck, shoulders and back are considered occupational health problems that may result in workers taking sick leave [1–4]. In Sweden, MSP is the second most common cause of work-related disorders [5], and studies have shown that the prevalence and incidence of sick leave due to long-term MSP (≥ 3 months) is high among women [2, 6]. According to a recent report from the Swedish Social Insurance Agency, 61% of Swedes who are on sick leave for MSP are women [5]. Furthermore, studies have shown that individuals on sick leave living with long-term MSP experience negative consequences in life, such as depression, social isolation and reduced income, which have been associated with their impaired work ability and reduced life satisfaction [7–9]. Life satisfaction, in the present study, is defined as satisfaction with all aspects of daily life [10–12] and work ability as a worker’s ability to manage his/her work tasks at a given point in time, in relation to his/her physical and psychosocial capacity [13, 14]. Considering the importance of a return to work (RTW) among women with MSP, it is essential to understand how work ability and life satisfaction contribute to the enabling of RTW.

Previous studies have shown that improved self-reported work ability has a positive impact on RTW in individuals with chronic MSP after a multidisciplinary rehabilitation program [15, 16]. Studies have also found that the self-reported work ability is associated with increased sustainable RTW among individuals with MSP [8]. However, it is unknown if work ability predicts RTW in women on sick leave due to long-term MSP. To the best of the authors’ knowledge, research about associations between life satisfaction and RTW among women with neck/shoulder and back pain are lacking. Earlier studies have largely focused on quality of life in relation to RTW among individuals with musculoskeletal disorders, particularly in rehabilitation settings [17–19].

The importance of work ability and life satisfaction for RTW has previously been studied in two systematic reviews among people with neck [20] and low back pain [20, 21]. Results from the reviews suggest that an individual’s expectations, personal beliefs and how they cope with their ability to work, are associated with RTW [20, 21]. Workplace factors such as high demands and low control at work (job strain) have also been shown to hinder workers in their RTW [22]. Some studies have reported that, among women, taking sick leave for MSP was more prevalent in male-dominated workplaces such as in technical occupations [23–25]. However, in Sweden, the statistics show that a majority of women who are on sick leave for MSP work in female-dominated workplaces such as health-, elderly- and child-care [26]. Since women and men tend to have different work tasks even in the same job, the rated work ability – and its relation to RTW – may differ between female populations and populations consisting of both men and women [27]. The instruments used in this study are the Work Ability Index (WAI) and Life Satisfaction Questionnaire (LiSat-11). The WAI is a commonly used instrument for assessing work ability [14] in relation to working life and rehabilitation. The LiSat-11 was originally developed for rehabilitation purposes [28]; in the present study, it was used to assess different aspects of life satisfaction among women on sick leave for long-term MSP. Previously, studies have investigated the predictive ability of the WAI particularly for sickness absence [29–31] and disability pension [32, 33] among both men and women. For sickness absence, the WAI could be used to identify people at risk of not returning to work in 2 weeks [29, 31]. However, van Schaaijk et al. [34] found that the WAI was unable to detect relevant changes after RTW among workers who had been on sick leave ≥2 weeks. Jääskeläinen et al. [32] showed that the WAI could be used to identify people who would receive disability pension. No studies concerning discriminative ability of the LiSat-11 for RTW and not RTW (NRTW) have been found. In this study, we aimed to investigate how well these instruments – the WAI and the LiSat-11 – are able to discriminate between RTW and NRTW among women on sick leave for MSP.

In summary, long-term MSP affects several aspects of daily life. For some women, it causes reduced ability to work and prolonged sick leave, and has negative consequences for their financial situation. Prolonged sick leave can also generate new problems related to inactivity and isolation, which in turn can lead to reduced life satisfaction and hinder RTW. To promote RTW in this population, it is important to determine what factors lead to RTW. Previous studies have mostly investigated the relationship between self-reported work ability and RTW in rehabilitation settings among both men and women. No studies have been found that investigate the relationship between life satisfaction, work ability and RTW among women with MSP. Therefore, the aim of this study was to determine whether work ability and life satisfaction could predict RTW among women with long-term neck/shoulder and/or back pain, and to assess the ability of the WAI and the LiSat-11 to discriminate between those who did RTW and those who did NRTW.

## Methods

### Study design and sample

This is a cohort study with 1-year follow-up, which is reported in accordance with STROBE guidelines [35]. In the spring of 2016, the Swedish Social Insurance Agency sent a baseline postal survey to all eligible women (*n* = 600) in central and northern Sweden who were currently receiving sick leave benefits from the Swedish Social Insurance Agency. Participants were recruited based on the medical certificate issued by their primary health care or hospital physician.

The inclusion criteria were women aged 18–65 years, ≥ 50% sick leave from their extent of employment, and duration of sick leave ≥1 month due to long-term (≥ 3 months) neck/shoulder and/or back pain. Neck/shoulder and/or back pain was classified according to the International Classification of Diseases-10 diagnostic codes: M53.1 (cervicobrachial syndrome), M54.2 (cervicalgia), M54.4 (lumbago with sciatica), M54.5 (low back pain), M54.9 (dorsalgia unspecified), M75.8 (other shoulder lesions), M75.9 (shoulder lesion, unspecified), and M79.1 (myalgia). Because the specific cause of MSP is often uncertain, many diagnosis codes are used for this population. The diagnosis codes were selected based on a previous study [36] and on discussions with the Swedish Social Insurance Agency. Understanding the Swedish language was also a requirement for the participants to be able to complete the questionnaire. Women were excluded from the study if they had been diagnosed with rheumatoid arthritis, multiple sclerosis, stroke, cancer, Parkinson’s disease, bipolar disease, schizophrenia, or pregnancy. These diseases, disorders and conditions were chosen as exclusion criteria, as individuals affected by them may have a different RTW process.

### Data collection

Prior to the selection procedure, two of the authors (MLK and AN) instructed personnel at the Swedish Social Insurance Agency on how to select participants. An initial invitation letter and a self-administered questionnaire including eight instruments were sent by the Swedish Social Insurance Agency to the participants. The self-administered questionnaires included the WAI [14] and the LiSat-11 [37]. Along with the questionnaire, a letter was sent providing information about the study and assuring the participants that their responses would be kept confidential. The participants gave their written informed consent together with the returned questionnaire. Two reminders were sent 2 weeks apart. A set of sociodemographic items asking about age, country of birth, cohabitation, education, years in the workforce, economic situation, life-long pain duration, pain intensity and type of work was also included. Physical activity was measured using a study specific question: How often do you exercise regularly for at least 30 min, e.g. walking, jogging, swimming, cycling or working in the garden? The four response alternatives were: 0 days/week, 1–3 days/week, 4–5 days/week, 6–7 days/week. A pain figure was also included to collect information on the location of pain on the body [38].

### Outcome variable

At the 1-year follow-up, the same questionnaire was sent to all participants who responded at baseline, with the addition of two questions to detect RTW status: “Are you working right now?” and “To what extent are you working?” If participants worked > 50% of their extent of employment at baseline, they were categorized as RTW; otherwise, they were categorized as NRTW.

### Explanatory variables

#### Work ability

The WAI [14] was used to measure work ability and reflects the individual’s perceptions about their present and immediate future ability to perform work with respect to work demands, health and mental resources. It consists of 7 items covering the individual’s: current work ability, work ability in relation to the physical and mental demands of the job, number of current diseases diagnosed by a physician, estimated impairment due to health status, sick leave over the past 12 months, self-prognosis of work ability in the next 2 years and mental resources. An index was made by summing up all single items. The total scores ranged from 7 to 49 points, with higher scores indicating greater work ability. In the present study, Cronbach’s α was 0.78.

#### Life satisfaction

Life satisfaction was measured using the LiSat-11 [37], which consists of 11 items. The 11 items cover different aspects of life: life as a whole, vocation, economy, leisure, contacts with friends and acquaintances, sexual life, activities of daily living, family life, partner relationship, physical and psychological health. Each item was rated on a 6-point ordinal scale ranging from 1 = very dissatisfied to 6 = very satisfied, with higher scores reflecting greater life satisfaction. The life satisfaction index was created by calculating the average score of the items, i.e. sum of item scores divided by the total number of items. In the present study, Cronbach’s α was 0.86.

### Covariates

The two covariates were “Type of work” and “Pain intensity”. Type of work was divided in white-collar, e.g., employees in office administration, nurses and teachers, and blue-collar, e.g., employees in elderly care, childcare and cleaning. Pain intensity was measured using three items of the Multidimensional Pain Inventory [39]: (i) How much pain are you experiencing right now? (ii) How much pain have you experienced on average during the past week? (iii) How much do you suffer from your pain? The participants rated each item on a 7-point Likert scale (0 = no pain; 6 = extreme pain). An index was created by calculating an average value of the items, with higher values indicating higher pain intensity. In the present study, Cronbach’s α was 0.76.

### Statistical analysis

Prior to the analyses, the normality of the data was assessed using scatterplots; all variables were approximately normally distributed. No outliers in the data were observed. Descriptive statistics are presented as proportions, means, and standard deviations.

To determine whether work ability and life satisfaction predict RTW, two multiple logistic regression analyses were conducted. In the unadjusted model, work ability and life satisfaction were considered simultaneously, and in the adjusted model, covariates were added. Because the number of participants allowed us to consider two covariates only [40], we selected type of work and pain intensity. The reasons for choosing these factors are that work ability depends on type of work [41] and that pain intensity has been found to be associated with work ability [42, 43]. Nagelkerke’s pseudo *R*^2^ was used as a measure of goodness-of-fit of the logistic regression model. Multi-collinearity between the predictors was examined using the variance inflation factor.

To assess the discriminative abilities of the WAI and the LiSat-11 regarding RTW and NRTW, receiver operating characteristic curves were fitted and the area under each curve was estimated using 95% confidence intervals. An area under curve value of 0.5 represents discrimination by chance, and a value of 1 is considered perfect discrimination [44]. Sensitivity and specificity for three specific cut-off points were derived from receiver operating characteristic curves: the lower and higher cut-off points had 95% sensitivity and specificity, respectively. The middle cut-off point was identified as the score with the maximum sum of sensitivity and specificity. Sensitivity represents true-positive rates, i.e., RTW was correctly identified as RTW, while specificity refers to true-negative rates, i.e., NRTW was correctly classified as NRTW. In all tests, the level of significance was set at *p* <  0.05. The statistical program SPSS (IBM, US) version 24 was used for all analyses.

## Results

Of the 600 women who received the questionnaire, 275 responded and 67 were excluded based on the exclusion criteria, giving a response rate of 39% (208/533 respondents), thus leaving 208 participants in the study at baseline. After 1 year (Spring 2017), a follow-up survey was sent to the 208 women who responded to the survey at baseline. The response rate was 68%, which corresponds to 141/208 respondents. An attrition analysis concerning age, work ability and life satisfaction was performed; it indicated no significant difference in mean values at baseline between participants and dropouts at follow-up. Table 1 presents the baseline characteristics of participants in the two groups: RTW and NRTW. Of the 141 women, 94 had RTW and 47 NRTW at the 1-year follow-up. At baseline, the mean work ability scores among women who had RTW and NRTW were approximately 26 and 18 points, respectively. The mean baseline scores for life satisfaction were 4.2 and 3.9 points for RTW and NRTW, respectively. At baseline, 57% of the women in the RTW group were on sick leave from full-time employment. Among the women in the NRTW group, 66% were on sick leave from full-time employment. At 1-year follow-up, women in the RTW group worked on average 87.5% of their extent of employment at baseline (SD = 16.5), while women in the NRTW group worked on average 9.1% (SD = 17.9).

**Table 1:** Baseline characteristics of participants who did RTW and who did NRTW after the 1-year follow-up

Variables	RTW (*n* = 94)	NRTW (*n* = 47)
Age (M, SD), years	49.04 ± 9.5	53.51 ± 8.3
Country of birth, n (%)		
Sweden	93 (98.9)	44 (93.6)
Others	1 (1.1)	3 (6.4)
Cohabitation, n (%)		
Living with partner	75 (79.8)	30 (63.8)
Living alone	17 (18.1)	12 (25.6)
Living apart	2 (2.1)	5 (10.6)
Education, n (%)		
Elementary	13 (13.8)	10 (21.3)
Upper secondary	45 (47.9)	22 (46.8)
University	32 (34.0)	14 (29.8)
Others	4 (4.3)	1 (2.1)
Years in the workforce^a^ (M, Range)	30.04 (6–46)	32.01 (3–47)
Duration of sick leave (M, SD), months	11.76 ± 17.2	35.63 ± 61.3
Economic situation, n (%)		
Very dissatisfied	5 (5.8)	9 (19.2)
Dissatisfied	19 (20.4)	8 (17.0)
Acceptable	36 (38.5)	21 (44.7)
Good	27 (28.7)	5 (10.6)
Very good	6 (6.6)	4 (8.5)
Life-long pain duration (M, Range), months	57.79 (3–264)	81.41 (4–264)
Pain intensity^b^ (M, SD)	3.70 ± 1.2	4.76 ± 0.8
Pain area, n (%)		
Neck/shoulders	65 (69.1)	32 (68.1)
Back	63 (67.0)	38 (80.9)
Neck/shoulders and back	36 (25.5)	25 (17.7)
Physical activity, n (%)		
0 day/week	12 (12.8)	7 (15.0)
1–3 days/week	48 (51.1)	16 (34.0)
4–5 days/week	24 (25.5)	12 (25.5)
6–7 days/week	10 (10.6)	12 (25.5)
Type of work^c^, n (%)		
White-collar	35 (37.2)	16 (34.0)
Blue-collar	59 (62.8)	31 (66.0)
Work ability^d^ (M, SD)	25.74 (7.4)	18.16 (6.0)
Well-being^e^ (M, SD)	4.20 (0.8)	3.93 (0.9)

*RTW* return to work, *NRTW* not return to work, *M* mean, *SD* standard deviation

^a^Total working years before going on sick leave

^b^Pain intensity measured using the Multidimensional Pain Inventory, scale 0–6 (higher values indicate higher pain intensity)

^c^Type of work (white-collar, e.g., employees in office administration, nurses and teachers, and blue-collar, e.g., employees in elderly care, childcare and cleaning)

^d^Work ability was measured using the WAI scale, where possible points range from 7 to 49

^e^Well-being was measured by the LiSat-11 scale, where possible points range from 1 to 6

### Work ability and life satisfaction

Table 2 indicates increased odds of RTW for women who rated high on work ability; the result remained significant after adjusting for type of work and pain intensity (OR 1.12, 95% CI: 1.04–1.22). Life satisfaction did not significantly predict RTW in the unadjusted or in the adjusted analyses. The variance inflation factor was less than 1.2, indicating no multi-collinearity between the independent variables in the prediction models [45]. The Nagelkerke’s pseudo *R*^2^ of the adjusted model was 0.32.

**Table 2:** Multiple logistic regression analyses of work ability and well-being as predictors of RTW at 1-year follow-up

Predictors	Unadjusted analysis			Adjusted analysis		
	OR	95% CI	*p-*value	OR	95% CI	*p-*value
Work ability	1.16	(1.07–1.25)	< 0.001	1.12	(1.04–1.22)	0.005
Well-being	1.01	(0.96–1.06)	0.68	1.01	(0.97–1.07)	0.58
Type of work^a^				1.25	(0.48–3.25)	0.65
Pain intensity				0.55	(0.34–0.93)	0.02

In the unadjusted model, work ability and life satisfaction were considered simultaneously, and in the adjusted model, covariates were added

*OR* odds ratio, *CI* confidence interval, *RTW* return to work

^a^Type of work = White collar (e.g., employees in office administration, nurses and teachers) with reference category blue-collar (e.g., employees in elderly care, childcare and cleaning)

In light of the findings for life satisfaction, additional logistic regression analyses were made using a single item from the LiSat-11 about satisfaction with the work situation instead of the full index covering all aspects of life. In the unadjusted analysis, only work ability predicted RTW (OR 1.16, 95% CI: 1.09–1.25). In the adjusted analysis, the results did not change (OR 1.12, 95% CI: 1.04–1.21), and pain intensity was significant (OR 0.51, 95% CI: 0.32–0.80).

### Discriminative ability of the WAI and the LiSat-11

Table 3 shows the ability of the WAI and the LiSat-11 at baseline to discriminate between RTW and NRTW. The WAI at baseline adequately discriminated between RTW and NRTW (Area under curve 0.78, 95% CI: 0.70–0.86). Using a cut-off point of 15, 95% of the women in the RTW group were correctly identified, while a cut-off point of 29 enabled 95% of the women in the NRTW group to be identified. The LiSat-11 at baseline did not significantly discriminate between RTW and NRTW.

**Table 3:** Area under the receiver operating characteristic curve for the WAI and the LiSat-11 at baseline. Sensitivity and specificity of the instruments for detecting RTW

	n	AUC	*p-*value	95% CI	Cut-off point	Sensitivity	Specificity
WAI^a^	207	0.78	< 0.001	0.70–0.86			
95% of sensitivity					15	0.95	0.34
Max (sensitivity + specificity)					23	0.66	0.77
95% of specificity					29	0.34	0.95
LiSat-11^b^	168	0.59	0.15	0.47–0.70			
95% of sensitivity					2.7	0.95	0.12
Max (sensitivity + specificity)					4.5	0.40	0.77
95% of specificity					5.4	0.07	0.95

WAI scale 7–49 points (higher values indicate greater work ability); LiSat-11 scale 1–6 points (higher values indicate greater well-being)

Using the cut-off points means that the values strictly below the cut-off point are classified as NRTW

*RTW* return to work, *AUC* Area under curve, *CI* Confidence Interval, *n* number of observations, *Max* the maximum value of the sensitivity and specificity

^a^*WAI* Work Ability Index

^b^*LiSat-11* Life Satisfaction questionnaire

## Discussion

The present results showed that work ability, but not life satisfaction, was able to predict RTW after 1 year among women with long-term neck/shoulder and/or back pain. Furthermore, the baseline WAI scores adequately discriminated between RTW and NRTW at the 1-year follow-up, while the baseline LiSat-11 scores did not.

The present results revealed an association between perceived work ability and RTW. This is in line with rehabilitation studies indicating that self-reported work ability increased the chance of RTW and sustainable RTW in women with MSP, including neck, shoulder and back pain [8, 15]. Similarly, previous studies have found that impaired work ability predicted future long-term sick leave, symptoms such as MSP and depression as well as poor health among women on sick leave for neck/shoulder pain [46, 47]. This indicates that work ability among women on sick leave for long-term MSP may play an important role not only for RTW, but also for their health. Considering the items in the WAI, the emphasis appears to be on work-related disability in relation to physical and psychosocial capacity. To facilitate RTW, healthcare staff could, for example, focus on reducing fear of physical activity and raising awareness of the benefits of being physically active. This may improve women’s ability to manage their work tasks in relation to physical and psychosocial capacity [13, 14].

After controlling for type of work and pain intensity, the results remained approximately the same. Higher pain intensity was associated with lower odds for RTW. This result is in accordance with previous studies on pain patients [48]. Larger studies are needed to elucidate the relationships between work ability, pain intensity and RTW in this population [43].

The present study showed that the WAI could be used to discriminate between women who RTW and NRTW after 1 year. Other studies have also found that the WAI has discriminate ability for sickness absence [29, 31], and disability pension [32]. The present findings suggested that the maximum combination of sensitivity and specificity was observed at the WAI cut-off point of 23 at baseline. For that cut-off point, specificity was higher, implying that women who eventually NRTW would be more correctly identified than women who eventually RTW. Naturally, the choice of the WAI cut-off point depends on the purpose of the screening. Sensitivity and specificity may not be equally important, and the appropriate cut-off point should be selected accordingly. For example, it may be relevant for healthcare providers to use the WAI cut-off point of 29 in rehabilitation to detect women at risk for NRTW. By identifying this group, preventive measures may be used more cost-effectively. Further testing of the cut-off points in new samples is needed before they can be recommended for use in screening for risk of NRTW in a rehabilitation setting.

It has previously been reported that women have many responsibilities at home, such as shopping and cleaning, which tend to cause them to combine part-time work with unpaid work, i.e. family responsibilities [49, 50]. This extended work may restrict their recovery from MSP, which may influence their work ability and life satisfaction, eventually influencing their RTW. In the present study, the instruments WAI and LiSat-11 were used because items in the WAI focus on the person’s physical and mental health and the ability to meet demands at work [13, 14], while the LiSat-11 focuses on satisfaction with aspects of daily life in general [11, 28]. With these two instruments, we took into account both the women’s work situation and their satisfaction with overall daily life, both of which could have an impact on RTW. However, the results showed that the LiSat-11 did not predict RTW, neither using the full index nor one item measuring the aspect of work. Furthermore, the LiSat-11 could not discriminate between RTW and NRTW. In a previous study, the EuroQol, an instrument measuring health-related quality of life, was able to predict RTW among individuals on sick leave for back and neck pain [18]. One reason for the difference in findings could be the focus of the LiSat-11 and EuroQol; while the LiSat-11 measures satisfaction of life as a whole, the EuroQol measures health-related quality of life [51]. Thus, it may be that this difference in focus makes the EuroQol a better instrument for screening. Further studies are required to determine whether other instruments measuring well-being/quality of life, e.g. the EuroQoL, are better able to discriminate between RTW and NRTW in this population of women. In addition, studies investigating associations between part time work, unpaid work and RTW are warranted.

### Strengths and limitations

A strength of the present study was the prospective design, which included a 1-year follow-up of 68% of the participants. Furthermore, we used inclusion criteria for participant selection that were based on International Classification of Diseases-10 codes provided by a physician. A limitation of the study was that the participants were not randomly selected. This could entail a possible sampling bias, which may affect the external validity of the study. Because the Swedish Social Insurance Agency invited the women to take part in the study, we had no access to non-respondents’ data. For this reason, a non-response analysis could not be performed. However, the characteristics of the participants show that their age distribution is similar to women on sick leave due to musculoskeletal pain in Sweden, and in other Nordic countries [2, 5]. During the follow-up period, we had no information about whether the participants had first returned to work and then relapsed to being on sick leave again, or whether the participants who were working to some extent at baseline had opportunities to receive support from the workplace and were, for this reason, more likely to be back at work compared to the participants who did not work at all. Moreover, information on whether or not the participants received treatment during the 1-year period was lacking. However, since people with MSP generally receive vocational rehabilitation in Sweden [5, 52], there is no reason to suspect that RTW and NRTW groups received different treatment during the follow-up period. Another potential limitation was the use of self-reported data to assess work ability, which may have caused common method bias in the results. In the present study, RTW was classified as working > 50% of their extent of employment at baseline, which may affect the generalizability of the results.

## Conclusions

This study supports a relationship between work ability and RTW among women on sick leave for long-term neck/shoulder and/or back pain. The results indicate that the WAI, but not the LiSat-11, can discriminate between RTW and NRTW in the population under study. Although the discriminative ability of the WAI needs to be verified in new samples before it can be recommended for use in rehabilitation settings, we suggest that healthcare professionals consider how women perceive their work ability in order to better support them in their RTW.

## Data Availability

The datasets used and analyzed during the present study are available from the corresponding author on reasonable request.

## Abbreviations

MSP: Musculoskeletal Pain
WAI: Work Ability Index
LiSat-11: Life Satisfaction Questionnaire
RTW: Return to Work
NRTW: Not RTW
